# Psychometric properties of an Arabic language version of the Treatment Adherence Questionnaire among dialysis patients with cardio renal syndrome

**DOI:** 10.3389/fpsyg.2026.1717948

**Published:** 2026-02-23

**Authors:** Adel Omar Laradhi, Mikiyas Amare Getu, Yan Shan, Abdulaziz Mansoor Al Raimi, Nahed Ahmed Hussien, Galal Albani, Mugahed Ali Al-khadher, Mohamed Elsayed Allawy

**Affiliations:** 1 Department of Medical Surgical Nursing, College of Nursing, University of Hail, Hail, Saudi Arabia; 2 The Third Affiliated Hospital of Zhengzhou University, Zhengzhou, Henan, China; 3 School of Nursing, Fudan University, Shanghai, China; 4 School of Nursing and Health, Woldia University, Weldiya, Ethiopia; 5 Seiyun Community College, Hadhramout, Yemen; 6 Faculty of Nursing, Suez Canal University, El Sheikh Zayed City, Egypt; 7 Maternal and Child Nursing Department, College of Nursing, University of Hail, Hail, Saudi Arabia; 8 Medical Surgical Nursing Department, Nursing College, Najran University, Najran, Saudia Arabia; 9 Department of Nursing Sciences, College of Applied Medical Sciences, Prince Sattam bin Abdulaziz University, Wadi ad-Dawasir, Saudi Arabia

**Keywords:** cardiorenal syndrome, dialysis, reliability, treatment adherence, validity

## Abstract

**Background:**

Assessing or measuring adherence is the initial step toward comprehending it, or the lack thereof.

**Objective:**

This study aims to translate and validate the Treatment Adherence Questionnaire TAQ among Yemeni dialysis patients with cardiorenal syndrome.

**Methods:**

This cross-cultural adaptation study was conducted at two dialysis centers in Hadhramout Governorate, Yemen, between January 10 and February 15 of 2021. The English version of TAQ was translated into Arabic using the Beaton translation process. Psychometric tests of the TAQ were performed on 100 patients with cardiorenal syndrome. For face and content validity, the pre-final version of the TAQ Arabic was tested using a convenience sample of 10 patients. Individual interviews were used to investigate the meaning, comprehensibility, and acceptability. To evaluate the factor loadings, discriminant validity, and internal consistency of the TAQ Arabic version (the TAQ-Ar), one hundred patients were selected in the same manner and using the same inclusion criteria. Exploratory Factor Analysis (EFA) was utilized to ascertain the underlying factor structure of the Arabic version of the questionnaire, as its dimensionality had not been previously determined in this group. We employed principal component extraction with promax rotation to produce a clear and understandable factor solution. To assess reproducibility, 30 patients were chosen randomly from 100 to resubmit the questionnaire 2 weeks after the initial administration and under the same conditions.

**Results:**

The transcultural adaptation process revealed that all TAQ-Ar items and patient descriptions exhibited full semantic equivalence. During the psychometric validation phase, the scale-level Content Validity Index was 0.88, and cognitive interviews (*n* = 30) confirmed patient clarity. Exploratory factor analysis identified four variables, aligning with the structure suggested by the original instrument inventor. Based on Bandalos' standards, factors were kept if their eigenvalues were more than 1 and their factor loadings were higher than 0.30. The overall average factor loading was 0.683, which shows that the test was valid. Internal consistency was very good (Cronbach's alpha = 0.948), and test-retest repeatability showed significant stability (ICC = 0.776).

**Conclusion:**

We confirm that the TAQ-Ar is suitable for assessing treatment adherence in individuals with Arabic as their first language for cardiorenal syndrome disease.

## 1 Background

A group of disorders known as cardiorenal syndrome (CRS) affects the heart and the kidneys. They can cause acute or chronic dysfunction in one organ that can cause acute or chronic dysfunction in the other (Rangaswami et al., 2019). CRS is a global problem that burdens the healthcare system significantly and contributes to mortality and morbidity (Savira et al., 2020). Complex interactions between neurohormones, inflammatory processes, oxidative stress, and abnormal metabolic processes make up the pathophysiology (Qiang et al., 2014). The management of CRS presents a challenge due to the intricate interactions between the heart and kidneys, as well as the potential for medications aimed at treating one organ's failure to exacerbate the function of the other organ (Cruz, 2013).

The World Health Organization (WHO) (2003) defines adherence as “the extent to which a person's behavior in taking medicine, following a diet, and implementing lifestyle changes corresponds with agreed-upon recommendations from a healthcare provider.” According to Ma et al. (2012) adherence is evaluated by asking participants if they adhere to each recommendation. It is defined as the person's willingness and ability to follow pharmacological or non-pharmacological intervention.

The effectiveness of renal replacement therapy depends on patients' adherence (Sousa et al., 2019) and persistence with the different components of the therapeutic strategy, which include a complex and challenging medication schedule involving various medications at different doses, numerous prescribed dialysis sessions with variable durations (Denhaerynck et al., 2007), dietary guidelines (Tsay, 2003; Kutner, 2001), and fluid restriction (Barnett et al., 2008). But as of right now, no method is considered to be the gold standard for measuring treatment adherence. Direct techniques cost more and are rarely utilized in clinical practice or scientific research, including giving active ingredients or their metabolites to patients via blood or urine (Castillo-Mancilla et al., 2016). In turn, indirect techniques, such as self-report scales, are more straightforward, less costly, and non-invasive (Osterberg and Blaschke, 2005; Gimenes et al., 2009). Psychometric analysis and scale-based cross-cultural validation can enhance the self-report adherence measure (Stirratt et al., 2015).

The wide variations in reported non-adherence rates, on the other hand, are primarily brought on by a shortage of reliable measurement tools that take into account the four standard components of treatment adherence behavior of patients with end-stage renal disease (ESRD) on maintenance hemodialysis (HD): attendance at HD sessions, taking prescribed medications, adhering to prescribed fluid restrictions, and dietary intake (Kim et al., 2010).

Assessing or measuring adherence is the initial step toward comprehending adherence, or the lack thereof. Healthcare providers and patients need a valid, reliable, and cost-effective method of measuring patient adherence to treatment in outpatient settings. The general use of such a tool will enhance our understanding of non-adherence and open the door for interventions targeted at improving adherence to therapies. It could provide light on modifiable factors impacting adherence in various patient populations (Morisky et al., 2008).

One of the few tools with demonstrated validity and reliability for evaluating treatment adherence in the ESRD population is the Dialysis Diet and Fluid Non-Adherence Questionnaire (DDFQ) (Vlaminck et al., 2001). The DDFQ evaluates the patients' adherence to diet and fluid restriction protocols over the preceding 14 days through four questions. However, adherence measurement is restricted since the DDFQ does not consider HD attendance and medication use (Vlaminck et al., 2001). The self-reported end-stage renal disease adherence questionnaire (ESRD-AQ) is a different tool designed to assess all aspects of dialysis patients' adherence behaviors, including restrictions on fluid, dietary, and prescription medication intake, as well as attendance at dialysis sessions (Kim et al., 2010). Although the ESRD-AQ instrument has 46 items and is divided into five sections, due to the absence of homogeneous items in the instrument's design, internal consistency reliability (Cronbach's α) has not been determined (Poveda et al., 2016).

One of the simple instruments used to measure the level of treatment adherence is the Treatment Adherence Questionnaire (TAQ). The original TAQ, developed and validated in 2015 by Ali Aminuddin Mohd Rasani in the English language, consists of 15 items and has been used to assess treatment adherence among patients with ESRD receiving hemodialysis (HD) in Malaysia (Rasani, 2015). The original version of the TAQ was developed in English and consists of 15 items. However, to date, the TAQ has not been translated or validated in any other languages or cultural contexts. This study is the first to attempt the translation and validation of the TAQ among Arabic-speaking patients. There is no measurement tool in Yemen or Arabic countries to evaluate the treatment adherence specific to hemodialysis patients. As a result, the study aimed to translate and validate the TAQ among Yemeni patients with a cardiorenal syndrome so that it could be used in Yemen to determine which patients are not adhering to treatment and, therefore, could benefit from interventions to reduce the risk of adverse events. In this context, our goal was to translate and validate the TAQ's Arabic version among Yemeni dialysis patients with cardiorenal syndrome.

## 2 Materials and methods

### 2.1 Design and setting

This cross-cultural adaptation study aimed to assess the validity and reliability of the Arabic version of the TAQ among Yemeni dialysis patients with cardiorenal syndrome. Permission for the cross-cultural adaptation was received from the developer of the TAQ (Rasani, 2015). The study was conducted in two dialysis centers in Hadhramout City, Yemen. Patients were enlisted during routine visits to the dialysis center between January 10 and February 15 of 2021.

### 2.2 Population and participants

The inclusion criteria were: patients diagnosed with cardiorenal syndrome for a minimum of 6 months, aged over 18 years, capable of reading and comprehending Arabic, and those who had undergone dialysis three times weekly for at least 3 months before the study's start. Patients who had a psychiatric or cognitive disorders were excluded. Patients were enrolled in the study using the convenience sampling technique. The item-respondent theory was used to determine the sample size. According to this theory, a ratio of 1:5 to 1:20 is suitable (Osborne and Costello, 2004; Dowrick et al., 2005; Williams et al., 2010). Therefore, a ratio of 1:5 was chosen, and the required sample size, based on the 15 questions of the questionnaire, was 75. The final sample size was 94, with a 20% non-response rate. But a larger sample size of 100 patients was enrolled to increase the reliability of the conclusion.

### 2.3 Ethical considerations

The studies involving humans were approved by the institutional review Board of Zhengzhou University (ZZUIRB, 2021-11). The studies were conducted in accordance with the local legislation and institutional requirements. The participants provided their written informed consent to participate in this study.

### 2.4 Material

The data were collected using a self-administered questionnaire, the tool consists of two parts. They are listed below:

#### 2.4.1 The Demographic and Clinical Data Questionnaire (DCDQ)

The Demographic and Clinical Data Questionnaire (DCDQ) was developed by the researcher and has two sections: (1) the first section has six questions to assess participants' demographic characteristics, including age, gender, marital status, educational level, occupation, and income. The second section, examining patient clinical data, had four questions in the form of short essays to assess patient clinical data containing questions related to CRS disease stage, duration of hemodialysis, comorbidities, and length of diagnosis with CRS.

#### 2.4.2 Treatment Adherence Questionnaire (TAQ)

The Treatment Adherence Questionnaire (TAQ) is a core questionnaire that is utilized to assess treatment adherence. A formal permission letter was obtained from Rasani (2015). This instrument includes four dimensions: dialysis adherence, medication adherence, fluid restriction adherence, and diet restriction adherence. The questionnaire contained 15 items with four dimensions, the first dimension of adherence against schedule HD has two items statement, the second dimension of adherence on drug consumption prescribed has 4 statement items, the third dimension of adherence with fluid restriction consists of 4 items statement, and the fourth dimension of adherence with dietary restrictions, consists of 5 items statement, and the response options ranged from 1 to 4 on a 4-point Likert scale (1 = never, 2 = sometimes, 3 = most of the time, 4 = all the time). The scores for negative statements were inverted. The total treatment adherence scores were computed by summing up the scores from items 1 to 15, with reverse scores of the items 2, 3, 6, 8, 10, 14, and 15. The range of possible scores is 15 to 60. A higher TAQ score indicates higher treatment adherence. The total scores are categorized into three: 15–29 (< 50%) indicated a low level of treatment adherence, 15–29 scores indicate low levels of treatment adherence; 30–44 scores indicate moderate levels of treatment adherence; and 45–60 scores indicate a high level of treatment adherence (Maharjan, 2016). The Cronbach alpha for the TAQ is 0.827, which can be regarded as an excellent value.

### 2.5 Adaptation process and psychometric evaluation of the instrument

#### 2.5.1 Phase 1 – cross-cultural adaptation

The Beaton et al. (2000) guidelines were used in the cross-cultural adaptation of the TAQ (Stirratt et al., 2015). Initially, two Arabic-native, bilingual translators independently created two English-to-Arabic translations and developed two forward translated versions (A and B). The two translators and the research leader compared the results of the two translations (A and B) and all minor issues encountered were addressed and resolved, as there were no major issues. Through consensus, a common forward translation (C) was synthesized. The Arabic version was then translated back into English by two English-native translators who were blind to the original English version. The TAQ's (Stirratt et al., 2015) original author was contacted for approval of the reverse translation. Semantic equivalence was decided upon by a committee made up of translators, an Arabic linguist, and questionnaire validation specialists. This equivalence signifies that the target language word and its equivalent in the source language convey the same meaning and nuance. The result indicates that both the Arabic and the original English translations have the same meaning for the corresponding words. Pretesting was done on 10 dialysis patients with cardiorenal syndrome (who were not included in the reliability and validity testing) to explore clarity, understandability, comprehensibility, and feasibility of the adapted version of the TAQ using a visual analog scale ranging from 0 (not clear at all and difficult to understand) to 10 (clear and easy to understand). The average score of 8.2 indicated that the adapted version was clear, comprehensible, and understandable. No ambiguity in the meaning of any item was reported. Thus, the pre-testing version was considered as a final version without any modification in the original English version, and the translation committee sent the final translation version to the principal investigator for approval and use.

#### 2.5.2 Phase 2 – psychometric evaluation

##### 2.5.2.1 Step 1: Face and content validity

**Figure 1:**
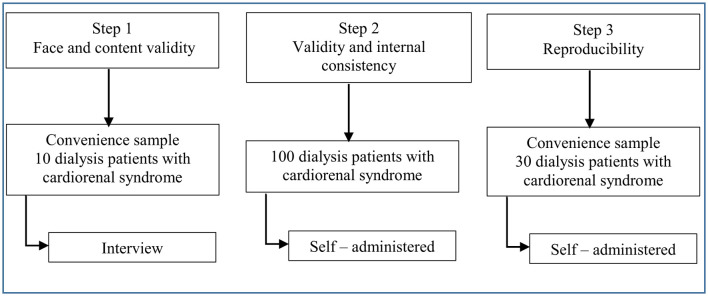
Procedure and steps for evaluating the psychometric properties of the Arabic version of the Treatment Adherence Questionnaire TAQ-Ar.

The pre-final version of the instrument was sent to seven experts to assess face and content validity of the instrument. An evaluation of the TAQ-Ar (Stirratt et al., 2015) pre-final version's face and content validity was conducted on a convenience sample of 10 Yemeni patients (Figure 1). During one-on-one interviews with the participants, the meaning, comprehensibility, and acceptability of each question's intended meaning were investigated.

##### 2.5.2.2 Step 2: Discriminant validity and internal consistency

One hundred Yemeni patients were recruited using the same criteria as in the first step. Both the internal consistency and discriminant validity of the TAQ-Ar were assessed.

To evaluate the instrument's underlying factor structure, an exploratory factor analysis (EFA) was carried out. The Kaiser–Meyer–Olkin (KMO) measure and Bartlett's test of sphericity were used to assess sampling adequacy and factorability before extraction. To obtain a more straightforward and comprehensible factor structure, factor extraction was carried out using the principal components method and Kaiser-Promax rotation. Items that showed factor loadings of at least 0.30 were kept, and factor retention decisions were based on eigenvalues larger than 1.

##### 2.5.2.3 Step 3: Reproducibility

Thirty patients were randomly chosen from the 100 participants in step 2 to complete the questionnaire a second time under identical conditions, with a duration of at least 14 days following the first administration to assess reproducibility with a 14-day interval. To examine the test-retest reliability, we employed the Intraclass Correlation Coefficient (ICC). A stable ICC was one that was 0.70 or above. The ICC value is 0.776.

Convenience sampling of one hundred (100) participants had given their consent and filled out the questionnaire. Yemeni patients diagnosed with cardiorenal syndrome disease for at least 6 months and aged 18 or older were eligible for inclusion. The ability to read and comprehend Arabic was a requirement for participation. Three times weekly dialysis for at least 3 months before the study begins.

After providing written consent, every eligible patient was personally approached and offered to participate. In Step 1, the interviews were conducted by a qualified research assistant. Participants were assured that their anonymity would be rigorously safeguarded and that only aggregated data would be disclosed. The confidentiality of participants was maintained. Face-to-face interviews were conducted to collect information from each participant. The interview was completed in no more than 20 min. Following the interview, the participant was requested to confirm that their response was accurate. The interviewer received training about active listening, minimizing personal bias, and developing a well-structured interview guide before data collection. Participants completed a self-administered questionnaire for steps 2 and 3 that included the TAQ-Ar, clinical information, and a sociodemographic profile. The sociodemographic characteristics including age, gender, marital status, educational level, income, occupation, number of family members, and daily diet prepared. The second section has four questions to assess the patients' clinical data. During the filling process, if there were any questions, the researchers answered them at any time. All questionnaires were collected on the spot and checked by the researchers. If they were incomplete, the patients were requested to supplement them in time. Dialysis centers were used to collect data via questionnaires (Figure 1).

### 2.6 Statistical analysis

To conduct statistical analysis, SPSS 21.0 for Windows was employed (SPSS, Inc., Chicago, IL). For a cross-cultural adaptation of the original Treatment Adherence Questionnaire (TAQ) into Arabic version the Treatment Adherence Questionnaire (TAQ), the percentage of agreement on semantic equivalence in patients for face and content validity was calculated. Internal consistency, reproducibility, factor analysis and discriminant properties were evaluated. Experts used intraclass correlation coefficients and item-level content validity indexes to validate and assess the TAQ-Ar, which consists of 15 items. To assess the degree of internal consistency, scores of items 2, 3, 6, 8, 10, 14, and 15 were reversed and Cronbach's alpha coefficients were calculated; Spearman correlation coefficients were used to measure the item-score correlations. As recommended, a Cronbach's α coefficient of 0.70 or greater is acceptable (Cortina, 1993). Reproducibility is a component of precision in a measurement system. It is the ability of a questionnaire to consistently reproduce the same measurement under the same conditions. It was assessed by repeating the administration of the Arabic version the Treatment Adherence Questionnaire (TAQ) to 30 subjects among the 100 who filled the questionnaire the first time, 2 weeks after the first administration. Intra-class correlation coefficients (ICC) with 95% confidence interval were calculated. ICC values ≥0.7 were considered acceptable. The Bartlett's sphericity tests were utilized (*p* < 0.0001), along with the Kaiser-Meyer-Olkin (KMO) index to assess the appropriateness of the sampling. Subsequently, a factor analysis was conducted using the principle components extraction method and the Kaiser-promax rotation method.

## 3 Results

### 3.1 Step 1: Face and content validity (*N* =10)

Thirty patients were involved in the pre-final evaluation of the TAQ-Ar, comprising 40% males, 60% individuals over 40 years of age, 80% married participants, and 70% with primary-level education. Cognitive individual interviews demonstrated complete semantic equivalence between the TAQ-Ar items and the paraphrases provided by patients; all participants indicated comprehension of the items and response options. Expert content validity was evaluated by an independent panel of seven experts who rated the relevance of each item using a 4-point scale (1 = not relevant to 4 = highly relevant). The scale-level content validity index (S-CVI/Ave) was 0.88, reflecting strong overall content validity. At the item level, the majority of items achieved an item-level content validity index (I-CVI) ≥ 0.78, meeting the recommended threshold criterion and demonstrating significant agreement among experts regarding the relevance of the instrument.

### 3.2 Step 2: Assessment of internal consistency (*N* = 100)

One hundred people were included in the study. The majority of patients (90%) were aged more than 40 years; the mean age of patients was 56.35 (SD = 13.26); more than half of patients (60%) were male. The majority of patients were married (80%). The highest percentage of patients (75%) had no formal education or had just completed elementary school, and the majority (95%) had insufficient monthly income. The sociodemographic and clinical characteristics are displayed in Table 1.

**Table 1:** Sociodemographic and clinical characteristics of participants (*N* = 100).

**Characteristics**	**Variable**	**Frequencies**	**Percentage %**
Gender	Male	60	60
	Female	40	40
Age	More than 40	90	90
	Less than 40	10	10
(mean ± SD)		(56.35 ± 13.26)	
Monthly income	Enough	5	5
	Not enough	95	95
Marital status	Not married	20	20
	Married	80	80
Occupation	Full-time	20	20
	Part-time	8	8
	Retired	6	6
	Unemployed	66	66
Education level	No formal education	30	30
	Primary school	45	45
	Secondary school	20	20
	Tertiary education or above	5	5
CRS disease stage	Stage 1	15	15
	Stage 2	5	5
	Stage 3	45	45
	Stage 4	35	35
Length of received hemodialysis	Less than 5	73	73
	5–10 years	18	18
	More than 10	9	9
Comorbidities	Other	19	19
	Diabetes mellitus	25	25
	Hypertension	56	56
Length of diagnosis with CRS	Less than 5	68	68
	5–10 years	23	23
	More than 10	9	9

CRS, Cardiorenal syndrome.

Participants' mean scores for the TAQ-Ar items are presented in Table 2, and the total mean score is 1.33 (0.18). The four subscales demonstrated strong internal consistency, with Cronbach's α ranging from 0.84 to 0.91, and excellent overall reliability. Test–retest correlations ranged from 0.65 to 0.72, while ICC values indicated acceptable stability across time (0.68–0.76). These findings support the reliability of all TAQ-Ar domains, including adherence to hemodialysis, medication, fluid restriction, and dietary restriction.

**Table 2:** Mean scores, internal consistency, test–retest reliability, and intra-class correlation of the Arabic Treatment Adherence Questionnaire (TAQ-Ar) subscales and items (*N* = 100).

**Domain/Item**	**Mean ±SD**	**Cronbach's α**	**Test–retest r**	**ICC (95% CI)**
Section A: Adherence to hemodialysis		0.88	0.68	0.71 (0.42–0.86)
I attended my dialysis treatment regularly.	2.95 ± 0.22			
I have shortened my dialysis time.	2.90 ± 0.44			
Section B: Adherence to medication		0.91	0.72	0.76 (0.50–0.88)
I missed the prescribed medications.	2.55 ± 0.67			
I took my medications even though I have problem due to side effect of the medications.	1.55 ± 0.81			
I took my prescribed medications even though I do not have any symptoms.	1.80 ± 1.03			
I stopped taking medication.	2.70 ± 0.72			
Section C: Adherence to fluid restriction		0.86	0.65	0.68 (0.39–0.82)
I followed the fluid restriction recommendation.	1.10 ± 0.95			
I took water as much as I want.	2.05 ± 0.81			
I managed my thirst, for example by staying in cool place, sipping my beverage, or using the ice cube.	1.25 ± 0.89			
I took food with hidden fluids, for example soup or ice creams.	2.15 ± 0.73			
Section D: Adherence to diet restriction		0.84	0.70	0.72 (0.48–0.86)
I followed the diet recommendation.	1.05 ± 0.87			
I took high protein foods, for example 2 matchbox size of meats, fish, or 1 drumstick of chicken every day.	1.60 ± 0.80			
I avoid foods containing salt.	0.50 ± 0.67			
I took high phosphate foods, for example beans, dried vegetables or fruits, or chocolate.	2.30 ± 0.56			
I took high potassium foods, for example bananas, papayas or oranges.	1.90 ± 0.63			

#### 3.2.1 Assessment of factor analysis and discriminant validity (*N* = 100)

The study was constrained to four factors, aligning with the factors originally proposed by the inventor of the instrument. Based on the criteria established by Bandalos and Finney (2010), the factors extracted in the instrument were determined based on eigenvalues greater than 1 and factor loadings over 0.30.

The average loading adherence to Hemodialysis was found to be 0.944, while the average loading adherence to Medication was 0.548. Additionally, the average loading adherence to fluid restriction was 0.650, and the average loading adherence to diet restriction was 0.590. These results indicate that discriminant validity has been established.

Table 3 shows the Arabic TAQ-Ar's factor structure and reliability indicators, which show that it works well as a psychometric tool. Exploratory factor analysis produced a four-factor solution corresponding to the original instrument domains: adherence to hemodialysis, medication, fluid restriction, and diet restriction, accounting for 65.6% of the variance, indicative of adequate construct representation. The factor loadings for the items varied from moderate to high (0.52–0.84), which shows that the items and factors were linked correctly. The items that were written negatively (marked^a^) also showed acceptable loading behavior. The Kaiser–Meyer–Olkin value (KMO = 0.805) proved that the sample was good enough, and Bartlett's test of sphericity (χ^2^ = 380.25, *p* < 0.001) showed that the factors could be found. The aggregate Cronbach's alpha for the 15 items was 0.948, which shows that there was a lot of internal consistency. The test-retest reliability was also strong (ICC = 0.776), which shows that it stays stable over time. All of these results support the Arabic TAQ-Ar's validity, reliability, and structural integrity as a tool for measuring treatment adherence.

**Table 3:** Factor structure, item–total correlation, internal consistency, and eigenvalues of the Arabic TAQ-Ar (*N* = 100).

**TAQ-Ar items**	**Factor 1**	**Factor 2**	**Factor 3**	**Factor 4**	**Eigenvalue**	**% Variance explained**
**Overall Cronbach's Alpha (15 items): 0.948**						
**Section A – Adherence to hemodialysis**						
I attended my dialysis treatment regularly.	0.80				4.30	28.7%
I have shortened my dialysis time	0.74					
**Section B – Adherence to medication**						
I missed the prescribed medications		0.70			2.60	17.3%
I took medications despite side effects.		0.68				
I took medication even when asymptomatic.		0.73				
I stopped taking medication		0.84				
**Section C – Adherence to fluid restriction**						
I followed fluid restriction recommendations.			0.71		1.85	12.3%
I drank water as much as I wanted			0.52			
I managed thirst (cool place, sipping, ice).			0.79			
I consumed foods with hidden fluids			0.58			
**Section D – Adherence to diet restriction**						
I followed the diet recommendations.				0.66	1.10	7.3%
I ate high-protein foods.				0.64		
I avoided salty foods.				0.60		
I consumed high-phosphate foods				0.69		
I consumed high-potassium foods				0.62		
Total variance	65.6%					

KMO = 0.805, Bartlett's χ^2^ = 380.25, df = 105, *p* < 0.001, and Test–retest ICC = 0.776.

### 3.3 Step 3: Assessment of reproducibility

Thirty subjects were studied, with 60% being male; 80% being over 40 years; 80% being married, and 70% have completed primary school or higher. Values of ICC greater than 0.776 which indicated indicated satisfactory reproducibility (Table 3).

#### 3.3.1 Reliability of the scale

Pretesting was conducted among respondents, during which scale mean, variance, item–total correlations, and Cronbach's alpha were assessed. All item-level alphas exceeded 0.70, and the overall scale demonstrated excellent reliability with a Cronbach's alpha of 0.948. Inter-item correlations further supported the internal consistency of the TAQ-Ar (Table 2).

## 4 Discussion

This study aimed to translate and develop an Arabic version of the TAQ and test its reliability and validity with patients on dialysis in Yemen who suffer from cardiorenal syndrome. Adapting a questionnaire for usage in another nation and language is time-consuming and costly. It is the best method for obtaining an equivalent metric for any self-attribute under consideration. Cross-cultural adaptation of self-administered health status questionnaires in new nations, languages, and/or cultures requires the use of unique approaches to establish equivalence between the original and target versions of the questionnaire (Beaton et al., 2000).

However, assessing adherence to prescription treatment among Arabic-speaking patients is difficult due to the lack of a validated Arabic version of the Treatment Adherence Questionnaire (TAQ) or other widely used and valid treatment adherence scales that may be administered for free (Lam and Fresco, 2015; Anghel et al., 2019; Ashur et al., 2015). As a result, the TAQ was validated and translated into Arabic, a language that more than 400 million people speak (Kamusella, 2017), which should make it easier to assess the treatment adherence of Arabic-speaking patients, especially those with chronic illnesses, which are widespread in the Arab world (Tyrovolas et al., 2020; Mokdad et al., 2014). So, this is the first study to our knowledge to validate an Arabic translation of the TAQ and to perform cross-cultural adaptation with a sample of Yemeni patients.

The methodology developed by the International Society of Quality of Life Assessment (IQOLA) was used in the research (Ware and Gandek, 1998; Ware et al., 1995). The TAQ was translated into Arabic and modified as part of the procedure covered in this paper to be usable and acceptable in a new cultural context. The process and testing detailed in this research demonstrated that translation does not inherently provide an accurate indicator of the health of another culture.

The patients responded positively to the questionnaire. It took 5 to 7 min to complete on average, and sometimes, patients could fill it out without much help. The TAQ-Ar demonstrated good psychometric properties in Yemeni dialysis patients with cardiorenal syndrome disease. The TAQ-Ar questions and the patient descriptions were completely semantically equivalent. A Cronbach's alpha of 0.948 indicated that the internal consistency was good, and it was 0.891 and 0.841 when items 6 and 9 were deleted, respectively.

The Arabic version of the TAQ was highly reliable. It was greater than the original (Rasani, 2015) and those reported from the Arabic and English versions of the GMAS in this sample, as well as the eight items of Morisky's Medication Adherence Scale (MMAS-8) (Ashur et al., 2015; AlShayban et al., 2020; Naqvi et al., 2020). One reason might be the homogenous distribution of our participants' features. This demonstrates the reliability and consistency of the scale.

## 5 Strengths and limitations

A significant strength of this study was the first validation study done globally on the TAQ, and the study used different reliability and validity tests. There were a few limitations to our study. Because a convenience sample was used, which raises concerns about the sample's representativeness. Inadequate sample size due to decreased patient flow during the COVID-19 pandemic period. Additionally, social desirability bias and reporting are possibilities because the study relied on self-reported questionnaires. Finally, it's possible that this study isn't representative of patients from other socioeconomic backgrounds because it focused on minority patients with meager incomes seeking routine dialysis care.

## 6 Implications

In clinical settings, the Arabic version of the Treatment Adherence Questionnaire is simple and easy to employ. The tool can identify patients who have problems adhering to treatment and track adherence throughout the treatment course.

## 7 Conclusion

The TAQ-Ar, which demonstrated acceptable reliability and validity, is a valid tool for assessing adherence in Yemeni-speaking cardiorenal syndrome patients, according to our findings. In clinical settings, the TAQ-Ar treatment adherence scale is simple and easy to employ. The tool can identify patients who have problems adhering to treatment and track adherence throughout the treatment course. One of the scale's most valuable features is its ability to quickly identify behavioral and attitude issues related to treatment, allowing medical staff to offer immediate support and guidance to patients. More studies should be done to validate this measurement scale in other settings and with other health problems.

## Data Availability

The data analyzed in this study is subject to the following licenses/restrictions: the data that support the findings of this study are available from the corresponding author upon reasonable request. Requests to access these datasets should be directed to abomalad2030@gmail.com.
