# Correction: Psychometric properties of an Arabic language version of the Treatment Adherence Questionnaire among dialysis patients with cardio renal syndrome

**DOI:** 10.3389/fpsyg.2026.1832186

**Published:** 2026-03-27

**Authors:** Adel Omar Laradhi, Mikiyas Amare Getu, Yan Shan, Abdulaziz Mansoor Al Raimi, Nahed Ahmed Hussien, Galal Albani, Mugahed Ali Al-khadher, Mohamed Elsayed Allawy

**Affiliations:** 1 Department of Medical Surgical Nursing, College of Nursing, University of Hail, Hail, Saudi Arabia; 2 The Third Affiliated Hospital of Zhengzhou University, Zhengzhou, Henan, China; 3 School of Nursing, Fudan University, Shanghai, China; 4 School of Nursing and Health, Woldia University, Weldiya, Ethiopia; 5 Seiyun Community College, Hadhramout, Yemen; 6 Faculty of Nursing, Suez Canal University, El Sheikh Zayed City, Egypt; 7 Maternal and Child Nursing Department, College of Nursing, University of Hail, Hail, Saudi Arabia; 8 Medical Surgical Nursing Department, Nursing College, Najran University, Najran, Saudia Arabia; 9 Department of Nursing Sciences, College of Applied Medical Sciences, Prince Sattam bin Abdulaziz University, Wadi ad-Dawasir, Saudi Arabia

**Keywords:** cardiorenal syndrome, dialysis, reliability, treatment adherence, validity

The funder “The Science and Technology Plan Research Project of Henan Provincial Department of Science and Technology”, 252102311148, was erroneously omitted.

The correct **Funding** statement reads:

The author(s) declared that financial support was received for this work and/or its publication. This project was supported by the Science and Technology Plan Research Project of Henan Provincial Department of Science and Technology, 252102311148.

The original version of this article has been updated.

